# An Enhanced Non-Coherent Pre-Filter Design for Tracking Error Estimation in GNSS Receivers

**DOI:** 10.3390/s17112668

**Published:** 2017-11-18

**Authors:** Zhibin Luo, Jicheng Ding, Lin Zhao, Mouyan Wu

**Affiliations:** College of Automation, Harbin Engineering University, Harbin 150001, China; zhaolin@hrbeu.edu.cn (L.Z.); wumouyan@hrbeu.edu.cn (M.W.)

**Keywords:** GNSS receivers, tracking error estimation, pre-filter design

## Abstract

Tracking error estimation is of great importance in global navigation satellite system (GNSS) receivers. Any inaccurate estimation for tracking error will decrease the signal tracking ability of signal tracking loops and the accuracies of position fixing, velocity determination, and timing. Tracking error estimation can be done by traditional discriminator, or Kalman filter-based pre-filter. The pre-filter can be divided into two categories: coherent and non-coherent. This paper focuses on the performance improvements of non-coherent pre-filter. Firstly, the signal characteristics of coherent and non-coherent integration—which are the basis of tracking error estimation—are analyzed in detail. After that, the probability distribution of estimation noise of four-quadrant arctangent (ATAN2) discriminator is derived according to the mathematical model of coherent integration. Secondly, the statistical property of observation noise of non-coherent pre-filter is studied through Monte Carlo simulation to set the observation noise variance matrix correctly. Thirdly, a simple fault detection and exclusion (FDE) structure is introduced to the non-coherent pre-filter design, and thus its effective working range for carrier phase error estimation extends from (−0.25 cycle, 0.25 cycle) to (−0.5 cycle, 0.5 cycle). Finally, the estimation accuracies of discriminator, coherent pre-filter, and the enhanced non-coherent pre-filter are evaluated comprehensively through the carefully designed experiment scenario. The pre-filter outperforms traditional discriminator in estimation accuracy. In a highly dynamic scenario, the enhanced non-coherent pre-filter provides accuracy improvements of 41.6%, 46.4%, and 50.36% for carrier phase error, carrier frequency error, and code phase error estimation, respectively, when compared with coherent pre-filter. The enhanced non-coherent pre-filter outperforms the coherent pre-filter in code phase error estimation when carrier-to-noise density ratio is less than 28.8 dB-Hz, in carrier frequency error estimation when carrier-to-noise density ratio is less than 20 dB-Hz, and in carrier phase error estimation when carrier-to-noise density belongs to (15, 23) dB-Hz ∪ (26, 50) dB-Hz.

## 1. Introduction

A Global Navigation Satellite System (GNSS) is a satellite system to provide autonomous positioning worldwide [1]. The representative instances of GNSS are the United States’ NAVSTAR Global Positioning System (GPS), the Russian GLONASS, the Chinese Beidou system (under deployment), and the European Union’s Galileo (under deployment) [2,3,4]. It allows electronic receivers that are smaller than a cell phone to determine their position, velocity and time (PVT) with high accuracy using signals transmitted by satellites anytime and anywhere around the globe. For instance, the B1I signal of the Beidou system—which offers open service—can provide a positioning accuracy of 10 meters (m), velocity accuracy of 0.2 meters per second (m/s), and timing accuracy of 50 nanoseconds (ns) with 95% confidence [5]. GNSS receivers have been widely used in military and civilian applications; for example, handset navigation, missile guidance, and aircraft landing [6,7,8]. It is a prevalent and significant research orientation to improve the navigation accuracy of GNSS receivers in harsh environments such as weak signal condition and highly dynamic scenario.

A classical GNSS signal is composed of a carrier, pseudo-random noise code (PRN code), and binary navigation message [9]. The GNSS receiver first estimates the PRN code phase, the phase of the carrier, and the carrier Doppler of the received GNSS signal. These parameters are referred to as GNSS signal parameters in the following. The estimation for GNSS signal parameters is done by the signal acquisition and tracking function of the GNSS receiver. The signal acquisition estimates GNSS signal parameters roughly in an opened-loop manner, while the signal tracking estimates these parameters accurately through closed-loop control [10]. A GNSS receiver must accomplish the signal acquisition before it switches to the signal tracking. After that, the GNSS receiver performs the measurement for pseudo-range, pseudo-range rate, and carrier phase according to the signal parameters estimated by signal tracking function. The physical meaning of pseudo-range and carrier phase is the relative distance between the GNSS receiver and navigation satellite, while the physical meaning of pseudo-range rate is the relative velocity between the receiver and the navigation satellite. The carrier phase, pseudo-range, and pseudo-range rate are referred to as GNSS measurement in the following. Meanwhile, the navigation message can also be extracted and demodulated to get the satellite ephemeris which can be used to calculate the position and velocity of navigation satellites. The above-mentioned signal processing—performed by GNSS receiver—can be referred to as the GNSS baseband signal processing. Finally, the GNSS receiver accomplishes the navigation computation to get the PVT parameters of the GNSS receiver itself through the GNSS measurements and GNSS satellite ephemeris. From the signal processing flow of GNSS receivers, it can be found that GNSS signal parameters estimation is the first step before the PVT solution can be obtained. Accurate signal parameters estimation is accomplished by tracking loops in the GNSS receiver. The tracking loops of the GNSS receiver include the carrier tracking loop and code tracking loop. Even though the tracking loops are able to accurately estimate the GNSS signal parameters, there still exits tracking error. The tracking error includes the carrier phase tracking error, carrier frequency tracking error, and code phase tracking error, which are referred to as carrier phase error, carrier frequency error, and code phase error for short in the following discussion. On the one hand, tracking error is the error signal of GNSS tracking loops that can be regarded as a kind of feedback control system. If the tracking error cannot be estimated accurately, it cannot be eliminated efficiently by the tracking loops. On the other hand, tracking error should be corrected in the GNSS measurement formulation phase. If the tracking error cannot be estimated precisely, the accuracy of GNSS measurements will decrease and the accuracy of PVT solution will subsequently decline. The tracking error estimation is especially crucial for low-cost GNSS receivers, because tracking error estimation of high accuracy will effectively improve the signal tracking ability and the accuracy of PVT determination. However, tracking error estimation is often corrupted by noise. That is because the tracking error is extracted from the noisy coherent integration and non-coherent integration, which are parts of GNSS baseband signal processing [11].

Generally, tracking error can be estimated by traditional discriminator or Kalman filter (KF)-based pre-filter. In terms of discriminator, there are a variety of discriminator algorithms that can be used for tracking error estimation, for example the two-quadrant arctangent (ATAN) discriminator for carrier phase error estimation, the four-quadrant arctangent (ATAN2) discriminator for carrier frequency error estimation, and non-coherent early minus late envelope (NC-EMLE) discriminator for code phase error estimation [9]. These discriminator algorithms are all implemented by the linear or nonlinear combination of coherent or non-coherent integration results. Unfortunately, there are some flaws for traditional discriminator. Firstly, the outputs of the traditional discriminator are often very noisy. That is, real tracking error is buried in noise. This situation becomes even worse in weak signal condition. Secondly, their effective working ranges are limited. For example, the most popular ATAN discriminator would only work properly when the input carrier phase error belongs to (−0.25 cycle, 0.25 cycle). If the carrier phase tracking error exceeds the scope of (−0.25 cycle, 0.25 cycle) (which may be caused by high dynamic motion), the ATAN discriminator would output the wrong carrier phase error estimation. Thirdly, not only the internal relationship among tracking error, but also the statistic characteristic of estimation noise of discriminator is totally ignored. Accurate tracking error estimation cannot be done by traditional discriminator.

Using KF-based pre-filter to estimate tracking error has recently received considerable attention in the design of GNSS scalar tracking loop, vector tracking loop, and GNSS/INS (i.e., Inertial Navigation System) deep integration navigation system that is of federated structure [12,13,14,15,16]. Compared with the traditional discriminator, the pre-filter would provide more accurate tracking error estimation because it fully utilizes the smoothing effect of the system model and the statistical characteristics of observation noise. The pre-filter in GNSS receivers can be classified into two categories: coherent pre-filter and non-coherent pre-filter [17]. The coherent pre-filter chooses coherent integration outputted by correlators as observations. For coherent pre-filter design, the observation vector is often a nonlinear function of state variables (i.e., tracking error), and hence it is often implemented by extended Kalman filter (EKF), which results in heavy computation burden. Many theoretical analyses have demonstrated that the coherent pre-filter can achieve high estimation accuracy in strong signal condition, but cannot perform well in weak signal condition [13]. However, there is no comprehensive performance evaluation of their performance in weak signal or high dynamic condition. The non-coherent pre-filter chooses discriminator outputs as observations. The system model and observation model of non-coherent pre-filter are all linear, thus a linear Kalman filter is enough for its implementation. It performs well in both strong signal and weak signal conditions. However, since it takes the discriminator outputs as observations, the performance of the non-coherent pre-filter is affected by drawbacks of the traditional discriminator. These drawbacks include the limited effective working region and unknown observation noise variance.

The coherent pre-filter and non-coherent pre-filter have complementary characteristics, which can be used to design a hybrid pre-filter structure [18]. An adaptive hybrid coherent/non-coherent GNSS vector tracking loop has been proposed to adaptively switch the pre-filter type that is working in tracking channel according to the signal strength [19]. The coherent pre-filter works in strong signal condition, while the non-coherent pre-filter works in weak signal condition. However, the switching threshold (24 dB-Hz) is determined by the designer’s experience, instead of comprehensive study.

This paper proposes an enhanced non-coherent pre-filter design and comprehensively evaluates the performance of coherent pre-filter, non-coherent pre-filter, and traditional discriminator. This paper is organized as follows: Section 2 provides the necessary basic knowledge about tracking error estimation, including the relevant baseband signal model, ATAN/ATAN2/NC-EMLE discriminator algorithms, and coherent/non-coherent pre-filter design. After that, Section 3 puts the emphasis on the observation noise statistics analysis and fault detection and exclusion (FDE) structure when designing the non-coherent pre-filter. In Section 4, four experiment scenes are designed to comprehensively evaluate the performance of enhanced non-coherent pre-filter. The performance of the enhanced non-coherent pre-filter is compared with that of the ATAN/ATAN2/NC-EMLE discriminator and coherent pre-filter to show the improvements. The best working conditions for the discriminator, non-coherent pre-filter, and coherent pre-filter are also analyzed in this section. Finally, Section 5 gives the conclusions and future work.

## 2. Tracking Error Estimation

This section first reviews some of the relevant baseband signal models that can be used in the tracking error estimation phase. In fact, the tracking error is extracted from these baseband signals. After that, three methods to estimate tracking error are introduced. They are ATAN/ATAN2/NC-EMLE discriminator, coherent pre-filter, and non-coherent pre-filter. This section is the basis of tracking error estimation.

### 2.1. Relevant Baseband Signal Model

**Figure 1:**
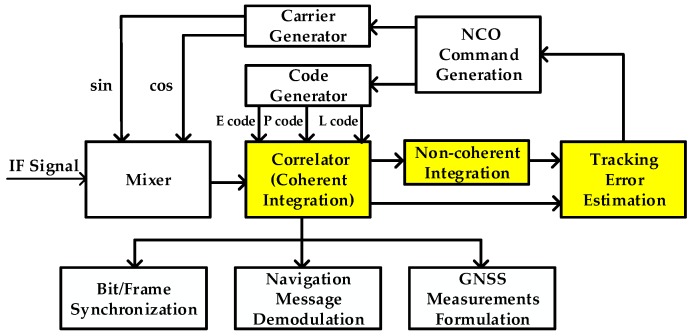
Baseband signal processing flow of GNSS receivers. IF: intermediate frequency; NCO: numerically-controlled oscillator.

Figure 1 illustrates the schematic block diagram of the baseband processing of GNSS receivers. The mixer, correlator, tracking error estimation model, numerically-controlled oscillator (NCO) command generation model, local carrier generator, and local PRN code generator constitute the basic GNSS tracking loop. The GNSS tracking loop, together with Bit/Frame synchronization model, navigation data demodulation model, and GNSS measurements formulation model constitute the intact baseband processing module. The whole baseband processing flow is described as follows.

After the GNSS signal passes through the radio frequency (RF) front end and is sampled, the center frequency of the digitalized signal is located in intermediate frequency (IF). As mentioned above, signal acquisition is first done to roughly estimate the GNSS signal parameters of all visible satellites. After that, GNSS tracking loops are employed to process the IF signal and accurately estimate GNSS signal parameters. The tracking loops include the carrier tracking loop and code tracking loop. The code tracking loop is often implemented as a delay lock loop (DLL), while the carrier tracking loop implemented as a phase lock loop (PLL), frequency lock loop (FLL), or FLL-assisted-PLL. In order to accomplish the GNSS signal tracking, the GNSS IF signal is first mixed with local generated sin/cos carrier signals with the aim of converting the signal center frequency from IF to baseband, and then correlated with local generated Early/Prompt/Late (E/P/L) PRN codes whose phase have certain interval to remove the PRN code modulated in GNSS signals. This correlation process can also be considered as coherent integration, which often contains the in-phase (I) component and quadrature (Q) component. Non-coherent integration can be further done by accumulating the auto-correlation amplitude or power which will be discussed later. The coherent/non-coherent integration contain the real tracking error. Thus, tracking error can be extracted from the coherent/non-coherent integration through traditional discriminator or pre-filter. After that, the NCO command generation model generates the carrier NCO and code NCO feedback command according to tracking error estimation, aiming at decreasing the tracking error. There are tracking loop algorithms in the NCO command generation model. Loop filter is the traditional method of generating the NCO feedback command [9,10,11]. The PLL, FLL, and DLL in this case all have similar structure in terms of loop filter design. Moreover, the Kalman filter-based tracking loop, the linear-quadratic-Gaussian (LQG)-based control method, and intelligent control method can also be used in the NCO command generation [20,21,22,23,24,25]. The other parts of baseband processing are Bit/Frame synchronization, navigation data demodulation, and GNSS measurements formulation, which are also shown in Figure 1, and are not discussed in detail in this paper. Based on the description above, it is coherent/non-coherent integration in baseband signal processing that are relevant to tracking error estimation. So, we will make a brief review of the mathematical model of coherent and non-coherent integration in the following part.

The analogy IF signal for all visible GNSS satellites is modeled as:

(1) $$s(t)=\sum_{i=1}^{N}a_{i}D_{i}(t)x_{i}(t)\cos[2\pi \cdot (f_{\mathrm{IF}}+f_{d,i})t+\varphi_{0,i}]+n_{0}(t)$$

where i indicates the i-th visible satellite, t is the signal reception time, a is signal amplitude, D is binary navigation data bits, x is the PRN code sequence, $f_{\mathrm{IF}}$ is analogy IF [Hz], $f_{d}$ is the Doppler frequency (Hz) caused by relative motion, $\varphi_{0}$ is the initial carrier phase (rad), $n_{0}(t)$ is additive band-limited white Gaussian noise. The one-sided power spectral density (PSD) of $n_{0}(t)$ is $N_{0}$ (Hz). The IF signal bandwidth depends on the RF front-end filter bandwidth which is no less than double the PRN code frequency. When the GNSS receiver tracks a specified GNSS signal in one tracking channel, other GNSS signals are suppressed and become noise because cross-correlation of different PRN codes is very little. So, the signal model for a specified GNSS satellite can be written as:

(2) $$s(t)=aD(t)x(t)\cos[2\pi \cdot (f_{\mathrm{IF}}+f_{d})t+\varphi_{0}]+n_{0}(t)$$

After sampled by the analog–digital converter (ADC) of the RF front-end, the signal becomes:

(3) $$s[m]=aD(mT_{s})x(mT_{s})\cos[2\pi \cdot (f_{\mathrm{IF}}+f_{d})mT_{s}+\varphi_{0}]+n_{0}(mT_{s})$$

where m is an integer and indicates the sampling point number, and $T_{s}$ is the sampling period (s) of the ADC.

The digitalized IF signal will first be mixed with local generated sin/cos carrier in mixers and then be correlated with local generated E/P/L PRN codes in correlators. The coherent integration can be obtained from the six correlators, shown as follows:

(4) $$\begin{array}{l} I_{E}(i)=A_{i}\cdot D_{i}\cdot R(\delta \tau_{i}-\frac{d}{2})\sin c(\delta f_{i}T_{\mathrm{coh}})\cos(\bar{\delta \varphi_{i}})+n_{\mathrm{IE}}\\ Q_{E}(i)=A_{i}\cdot D_{i}\cdot R(\delta \tau_{i}-\frac{d}{2})\sin c(\delta f_{i}T_{\mathrm{coh}})\sin(\bar{\delta \varphi_{i}})+n_{\mathrm{QE}}\\ I_{P}(i)=A_{i}\cdot D_{i}\cdot R(\delta \tau_{i})\sin c(\delta f_{i}T_{\mathrm{coh}})\cos(\bar{\delta \varphi_{i}})+n_{\mathrm{IP}}\\ Q_{P}(i)=A_{i}\cdot D_{i}\cdot R(\delta \tau_{i})\sin c(\delta f_{i}T_{\mathrm{coh}})\sin({\bar{\delta \varphi }}_{i})+n_{\mathrm{QP}}\\ I_{L}(i)=A_{i}\cdot D_{i}\cdot R(\delta \tau_{i}+\frac{d}{2})\sin c(\delta f_{i}T_{\mathrm{coh}})\cos(\bar{\delta \varphi_{i}})+n_{\mathrm{IL}}\\ Q_{L}(i)=A_{i}\cdot D_{i}\cdot R(\delta \tau_{i}+\frac{d}{2})\sin c(\delta f_{i}T_{\mathrm{coh}})\sin({\bar{\delta \varphi }}_{i})+n_{\mathrm{QL}} \end{array}$$

where i indicates the i-th coherent integration results, $I_{E}$, $Q_{E}$, $I_{P}$, $Q_{P}$, $I_{L}$, $Q_{L}$ are coherent integration in the six correlators, $\sin c(\delta fT_{\mathrm{coh}})=\sin(\pi \delta fT_{\mathrm{coh}})/\pi \delta fT_{\mathrm{coh}}$, $T_{\mathrm{coh}}$ is coherent integration time (s), δτ denotes the code phase error (chip), δf denotes the carrier frequency error (Hz), $\bar{\delta \varphi }$ denotes the mean carrier phase error (rad) in coherent integration time, R(⋅) denotes the auto-correlation function of PRN code, d denotes the Early-Late correlator spacing (chip), $n_{\mathrm{IE}}$, $n_{\mathrm{QE}}$, $n_{\mathrm{IP}}$, $n_{\mathrm{QP}}$, $n_{\mathrm{IL}}$, $n_{\mathrm{QL}}$ represent the noise in coherent integration. It should be noted here that the distributions of $n_{\mathrm{IE}},\;n_{\mathrm{QE}},\;n_{\mathrm{IP}},\;n_{\mathrm{QP}},\;n_{\mathrm{IL}},\;n_{\mathrm{QL}}$ almost fulfill the additive white Gaussian noise (AWGN) assumption (i.e., $n_{\mathrm{IE}},\;\ldots ,\;n_{\mathrm{QL}}\sim N(0,\sigma_{\mathrm{noise}}{}^{2})$). The value of A can be calculated by the following Equation (5) [9]:

(5) $$A=\sqrt{2(c/n_{0})T_{\mathrm{coh}}}\sigma_{\mathrm{noise}}$$

where $c/n_{0}$ is the carrier-to-noise density ratio (Hz) of the corresponding GNSS signal, $\sigma_{\mathrm{noise}}$ is the standard deviation of $n_{\mathrm{IE}},\;n_{\mathrm{QE}},\;n_{\mathrm{IP}},\;n_{\mathrm{QP}},\;n_{\mathrm{IL}},\;n_{\mathrm{QL}}$.

After coherent integration is done, the auto-correlation power and auto-correlation amplitude can be defined as Equations (6) and (7), respectively:

(6) $$\begin{array}{ll} E^{2}(i)=I_{E}{}^{2}(i)+Q_{E}{}^{2}(i)=A_{i}{}^{2}R^{2}(\delta \tau_{i}-\frac{d}{2}){\sin c}^{2}(\delta f_{i}T_{\mathrm{coh}}) & +n_{E^{2}}\\ P^{2}(i)=I_{P}{}^{2}(i)+Q_{P}{}^{2}(i)=A_{i}{}^{2}R^{2}(\delta \tau_{i}){\sin c}^{2}(\delta f_{i}T_{\mathrm{coh}}) & +n_{P^{2}}\\ L^{2}(i)\;=I_{L}{}^{2}(i)+Q_{L}{}^{2}(i)=A_{i}{}^{2}R^{2}(\delta \tau_{i}+\frac{d}{2}){\sin c}^{2}(\delta f_{i}T_{\mathrm{coh}}) & +n_{L^{2}} \end{array}$$

(7) $$\begin{array}{ll} E(i)=\sqrt{I_{E}{\left(i\right)}^{2}+Q_{E}{\left(i\right)}^{2}}=A_{i}\cdot R(\delta \tau_{i}-\frac{d}{2})\left|\sin c(\delta f_{i}T_{\mathrm{coh}})\right|\; & +n_{E}\\ P(i)=\sqrt{I_{P}{}^{2}(i)+Q_{P}{}^{2}(i)}=A_{i}\cdot R(\delta \tau_{i})\left|\sin c(\delta f_{i}T_{\mathrm{coh}})\right| & +n_{P}\\ L(i)\;=\sqrt{I_{L}{}^{2}(i)+Q_{L}{}^{2}(i)}=A_{i}\cdot R(\delta \tau_{i}+\frac{d}{2})\left|\sin c(\delta f_{i}T_{\mathrm{coh}})\right|\; & +n_{L} \end{array}$$

where

(8) $$\begin{array}{ccc} n_{E^{2}} & =n_{\mathrm{IE}}{}^{2}+n_{\mathrm{QE}}{}^{2}+2n_{\mathrm{IE}}A_{i}\cdot D_{i}\cdot R(\delta \tau_{i}-\frac{d}{2})\sin c(\delta f_{i}T_{\mathrm{coh}})\cos(\bar{\delta \varphi }) & +\\ & 2n_{\mathrm{QE}}A_{i}\cdot D_{i}\cdot R(\delta \tau_{i}-\frac{d}{2})\sin c(\delta f_{i}T_{\mathrm{coh}})\sin(\bar{\delta \varphi }) & \\ & \approx n_{\mathrm{IE}}{}^{2}+n_{\mathrm{QE}}{}^{2}+n_{\mathrm{IE}} & \\ n_{P^{2}} & =n_{\mathrm{IP}}{}^{2}+n_{\mathrm{QP}}{}^{2}+2n_{IP}A_{i}\cdot D_{i}\cdot R(\delta \tau_{i}-\frac{d}{2})\sin c(\delta f_{i}T_{\mathrm{coh}})\cos(\bar{\delta \varphi }) & +\\ & 2n_{\mathrm{QP}}A_{i}\cdot D_{i}\cdot R(\delta \tau_{i}-\frac{d}{2})\sin c(\delta f_{i}T_{\mathrm{coh}})\sin(\bar{\delta \varphi }) & \\ & \approx n_{\mathrm{IP}}{}^{2}+n_{\mathrm{QP}}{}^{2}+n_{\mathrm{IP}} & \\ n_{L^{2}} & =n_{\mathrm{IL}}{}^{2}+n_{\mathrm{QL}}{}^{2}+2n_{\mathrm{IL}}A_{i}\cdot D_{i}\cdot R(\delta \tau_{i}+\frac{d}{2})\sin c(\delta f_{i}T_{\mathrm{coh}})\cos(\bar{\delta \varphi }) & +\\ & 2n_{\mathrm{QL}}A_{i}\cdot D_{i}\cdot R(\delta \tau_{i}+\frac{d}{2})\sin c(\delta f_{i}T_{\mathrm{coh}})\sin(\bar{\delta \varphi }) & \\ & \approx n_{\mathrm{IL}}{}^{2}+n_{\mathrm{QL}}{}^{2}+n_{\mathrm{IL}} & \end{array}$$

(9) $$\begin{array}{ll} n_{E} & =\sqrt{A_{i}{}^{2}R^{2}(\delta \tau_{i}-\frac{d}{2}){\sin c}^{2}(\delta f_{i}T_{\mathrm{coh}})+n_{E^{2}}}-A\cdot R(\delta \tau_{i}-\frac{d}{2})\left|\sin c(\delta f_{i}T_{\mathrm{coh}})\right|\\ & \approx \sqrt{A_{i}{}^{2}{\left(1-\frac{d}{2}\right)}^{2}+n_{E^{2}}}-A_{i}(1-\frac{d}{2})\\ n_{P} & =\sqrt{A_{i}{}^{2}R^{2}(\delta \tau_{i}){\sin c}^{2}(\delta f_{i}T_{\mathrm{coh}})+n_{P^{2}}}-A\cdot R(\delta \tau_{i})\left|\sin c(\delta f_{i}T_{\mathrm{coh}})\right|\\ & \approx \sqrt{A_{i}{}^{2}+n_{P^{2}}}-A_{i}\\ n_{L} & =\sqrt{A_{i}{}^{2}R^{2}(\delta \tau_{i}+\frac{d}{2}){\sin c}^{2}(\delta f_{i}T_{\mathrm{coh}})+n_{L^{2}}}-A_{i}\cdot R(\delta \tau_{i}+\frac{d}{2})\left|\sin c(\delta f_{i}T_{\mathrm{coh}})\right|\\ & \approx \sqrt{A_{i}{}^{2}{\left(1-\frac{d}{2}\right)}^{2}+n_{L^{2}}}-A_{i}(1-\frac{d}{2}) \end{array}$$

It should be noted that the tracking loops are assumed to be in locking state when we derive the Equations (8) and (9). That is, tracking error is assumed to be zero. After that, the non-coherent integration can be obtained by accumulating the auto-correlation amplitude of size $N_{\mathrm{nc}}$, as shown in Equation (10). Another form of non-coherent integration is calculated by accumulating the auto-correlation power of size $N_{\mathrm{nc}}$, as shown in Equation (11). The carrier frequency error and signal amplitude are assumed to be constant when we derive Equations (10) and (11).

(10) $$\begin{array}{l} E=\sum_{j=1}^{N_{\mathrm{nc}}}E(j)=N_{\mathrm{nc}}A\left|\sin c(\delta fT_{\mathrm{coh}})\right|\;\sum_{j=1}^{N_{nc}}R(\delta \tau_{j}-\frac{d}{2})+N_{\mathrm{nc}}n_{E}\\ P=\sum_{j=1}^{N_{\mathrm{nc}}}P(j)=N_{\mathrm{nc}}A\left|\sin c(\delta fT_{\mathrm{coh}})\right|\;\sum_{j=1}^{N_{nc}}R(\delta \tau_{j})+N_{\mathrm{nc}}n_{P}\\ L\;=\sum_{j=1}^{N_{\mathrm{nc}}}L(j)=N_{\mathrm{nc}}A\left|\sin c(\delta fT_{\mathrm{coh}})\right|\;\sum_{j=1}^{N_{nc}}R(\delta \tau_{j}+\frac{d}{2})+N_{\mathrm{nc}}n_{L} \end{array}$$

(11) $$\begin{array}{l} E^{2}=\sum_{j=1}^{N_{\mathrm{nc}}}E^{2}(n)=N_{\mathrm{nc}}A^{2}{\left|\sin c(\delta fT_{\mathrm{coh}})\right|}^{2}\;\sum_{j=1}^{N_{nc}}R^{2}(\delta \tau_{j}-\frac{d}{2})+N_{\mathrm{nc}}n_{E^{2}}\\ P^{2}=\sum_{j=1}^{N_{\mathrm{nc}}}P^{2}(n)=N_{\mathrm{nc}}A^{2}{\left|\sin c(\delta fT_{\mathrm{coh}})\right|}^{2}\;\sum_{j=1}^{N_{nc}}R^{2}(\delta \tau_{j})+N_{\mathrm{nc}}n_{P^{2}}\\ L^{2}\;=\sum_{j=1}^{N_{\mathrm{nc}}}L^{2}(n)=N_{\mathrm{nc}}A^{2}{\left|\sin c(\delta fT_{\mathrm{coh}})\right|}^{2}\;\sum_{j=1}^{N_{nc}}R^{2}(\delta \tau_{j}+\frac{d}{2})+N_{\mathrm{nc}}n_{L^{2}} \end{array}$$

Equations (4), (10), and (11) are exactly the baseband signal models that can be used for tracking error estimation. These baseband signal models are summarized in Table 1, where they are expressed as the addition of useful signal and noise. The useful signal contains the real tracking error, and the noise effect cannot be cancelled when the tracking error is estimated using these baseband signals. We also make a brief comment about the probability distribution of noise in this table.

### 2.2. Traditional Discriminator

There is a variety of discriminator algorithms for tracking error estimation. The ATAN discriminator and ATAN2 discriminator are the research objects for carrier phase error and carrier frequency error estimation in this paper. Because they are optimal (maximum likelihood estimator) at both high and low signal to noise ratio (SNR) and their slopes are signal amplitude dependent [11]. As for code phase error estimation, the most popular NC-EMLE discriminator is the research object.

The ATAN, ATAN2, and NC-EMLE discriminator algorithms are expressed in Equations (12)–(14), respectively, as follows:

(12) $${\bar{\delta \varphi }}_{\mathrm{ATAN}}=\arctan\frac{Q_{P}}{I_{P}}$$

(13) $$\delta f_{\mathrm{ATAN}2}=\frac{\arctan2(P_{\mathrm{cross}},P_{\mathrm{dot}})}{2\pi \cdot T_{\mathrm{coh}}}$$

(14) $$\delta \tau_{\mathrm{NC}-\mathrm{EMLE}}=(1-d)\frac{E-L}{E+L}$$

where ${\bar{\delta \varphi }}_{\mathrm{ATAN}}$ is carrier phase error estimation (cycle), $\delta f_{\mathrm{ATAN}2}$ is carrier frequency error estimation (Hz), $\delta \tau_{\mathrm{NC}-\mathrm{EMLE}}$ is code phase error estimation (chip), the definitions of $P_{\mathrm{cross}},\;P_{\mathrm{dot}}$ are shown in Appendix A. Plug the mathematical models of coherent/non-coherent integration, which have been shown in Table 1, into the Equations (12)–(14), these discriminator algorithms can be reformulated as:

(15) $${\bar{\delta \varphi }}_{\mathrm{ATAN}}=\arctan\frac{A\cdot D\cdot R(\delta \tau )\sin c(\delta fT_{\mathrm{coh}})\sin(\bar{\delta \varphi })+n_{\mathrm{QP}}}{A\cdot D\cdot R(\delta \tau )\sin c(\delta fT_{\mathrm{coh}})\cos(\bar{\delta \varphi })+n_{\mathrm{IP}}}=\bar{\delta \varphi }+n_{\mathrm{ATAN}}$$

(16) $$\delta f_{\mathrm{ATAN}2}=\frac{1}{T_{\mathrm{coh}}}\arctan\frac{B(n)B(n-1)\sin(\delta \varphi_{n}-\delta \varphi_{n-1})+C_{1}(n)}{B(n)B(n-1)\cos(\delta \varphi_{n}-\delta \varphi_{n-1})+C_{2}(n)}=\delta f+n_{\mathrm{ATAN}2}$$

(17) $$\delta \tau_{\mathrm{NC}-\mathrm{EMLE}}=(1-d)\frac{A\left|\sin c(\delta fT_{coh})\right|\cdot \{\sum_{j=1}^{N_{nc}}R(\delta \tau -\frac{d}{2})-\sum_{j=1}^{N_{nc}}R(\delta \tau_{j}+\frac{d}{2})\}+N_{\mathrm{nc}}n_{E}-N_{\mathrm{nc}}n_{L}}{A\left|\sin c(\delta fT_{\mathrm{coh}})\right|\cdot \{\sum_{j=1}^{N_{nc}}R(\delta \tau -\frac{d}{2})+\sum_{j=1}^{N_{nc}}R(\delta \tau_{j}+\frac{d}{2})\}+N_{\mathrm{nc}}n_{E}+N_{\mathrm{nc}}n_{L}}=\delta \tau +n_{\mathrm{NC}-\mathrm{EMLE}}$$

where B(n), $C_{1}(n)$, $C_{2}(n)$ are derived and defined in Appendix A, and $n_{\mathrm{ATAN}}$, $n_{\mathrm{ATAN}2}$, and $n_{\mathrm{NC}-\mathrm{EMLE}}$ are estimation noise of corresponding discriminator. It should be noted here that the discriminator outputs are expanded in the form of the summation of real tracking error and estimation noise in Equations (15)–(17). Thus, it is clear that outputs of ATAN/ATAN2/NC-EMLE discriminator contain the real tracking error and estimation noise (i.e., $n_{\mathrm{ATAN}}$, $n_{\mathrm{ATAN}2}$, and $n_{\mathrm{NC}-\mathrm{EMLE}}$). These discriminator algorithms make non-linearity processing for useful signal and noise of coherent/non-coherent integration (e.g., division and arctangent operation). Thus it is difficult to get the analytic solution for the probability distribution of estimation noise, which is the barrier to implementing the non-coherent pre-filter.

The estimation noise variance of the ATAN discriminator has been derived in [10], with some necessary simplification, and is shown in Equation (18). The estimation noise variance of the ATAN2 discriminator is derived in Appendix B based on Equation (18), and the result is shown in Equation (19). However, the accuracy of variance estimation of Equations (18) and (19) would decrease with decreasing *C*/*N*_0_, as will be shown in Section 3.1. Besides, the estimation noise variance of the NC-EMLE discriminator is difficult to derive.

(18) $$\sigma_{\mathrm{ATAN}}{}^{2}=D(n_{\mathrm{ANAN}})={\left(\frac{1}{2\pi }\right)}^{2}\frac{1}{2\cdot c/n_{0}\cdot T_{\mathrm{coh}}}(1+\frac{1}{c/n_{0}\cdot T_{\mathrm{coh}}}){[\mathrm{cycle}}^{2}]$$

(19) $$\sigma_{\mathrm{ATAN}2}{}^{2}=D(n_{\mathrm{ANAN}2})={\left(\frac{1}{2\pi }\right)}^{2}\frac{1}{c/n_{0}\cdot T_{\mathrm{coh}}{}^{3}}(1+\frac{1}{c/n_{0}\cdot T_{\mathrm{coh}}})[{\mathrm{Hz}}^{2}]$$

### 2.3. Non-Coherent Pre-Filter Design

The non-coherent pre-filter is based on a linear Kalman filter. The state variables of non-coherent pre-filter are code phase error (chip), carrier phase error (cycle), Doppler frequency error (Hz), and Doppler frequency rate error (Hz/s), respectively. The state variables of non-coherent pre-filter are shown in the following equation:

(20) $$X_{\mathrm{nonCoh}}={\left[\delta \tau \;\delta \varphi \;\delta f\;\dot{\delta }f\right]}^{T}$$

The state transition matrix is constructed by assuming a constant acceleration model for carrier phase error and a constant position model for code phase error. In this transition matrix, the carrier-aiding for DLL is used so that dynamic stress for DLL is overcome by PLL. Thus, the narrow bandwidth DLL can be used to decrease the code tracking error. The inherent relationship between state variables is fully exploited. The state transition matrix is expressed as follows:

(21) $$A_{\mathrm{nonCoh}}=\left[\begin{array}{cccc} 1 & 0 & \beta T_{\mathrm{coh}} & 0\\ 0 & 1 & T & \frac{T_{\mathrm{coh}}{}^{2}}{2}\\ 0 & 0 & 1 & T_{\mathrm{coh}}\\ 0 & 0 & 0 & 1 \end{array}\right]$$

where β is the scale factor converting the carrier Doppler frequency to code chip rate.

The process noise vector $w_{1}$ of non-coherent pre-filter is given by:

(22) $$w_{1}={\left[w_{\mathrm{code}}\;w_{\mathrm{clock}}\;w_{\mathrm{drift}}\;w_{\mathrm{accel}}\right]}^{T}$$

where $w_{\mathrm{code}}$ is the process noise for the code phase error to account for code multipath effects, $w_{\mathrm{clock}}$ is process noise for the clock bias, $w_{\mathrm{drift}}$ is the process noise for the clock drift, and $w_{\mathrm{accel}}$ is the process noise for the phase acceleration (which is related to the receiver dynamics) [23]. The corresponding covariance matrix of system model for coherent pre-filter is given by

(23) $$Q_{\mathrm{nonCoh}}=E[w_{1}w_{1}{}^{T}]$$

The ATAN/ATAN2/NC-EMLE discriminator outputs are directly used as observation vector, shown as

(24) $$Z_{\mathrm{nonCoh}}={\left[\delta \tau_{\mathrm{NC}-\mathrm{EMLE}}\;\delta \varphi_{\mathrm{ATAN}}\;\delta f_{\mathrm{ATAN}2}\right]}^{T}$$

According to Equations (15)–(17), it is obvious that the observation matrix can be given in a very simple form as

(25) $$C_{\mathrm{nonCoh}}=\left[\begin{array}{cccc} 1 & 0 & 0 & 0\\ 0 & 1 & 0 & 0\\ 0 & 0 & 1 & 0 \end{array}\right]$$

### 2.4. Coherent Prefilter Design

The coherent pre-filter is based on EKF because of the non-linearity of the observation model. In this case, the correlator outputs are used directly to estimate the signal amplitude, initial carrier phase error, initial carrier frequency error, and initial carrier frequency rate error (“initial” in this context refers to the beginning of coherent integration interval). The state vector and observation vector of coherent pre-filter are shown as shown in Equations (26) and (27), respectively.

(26) $$X_{\mathrm{coh}}={\left[A\;\delta \tau \;\delta \varphi \;\delta f\;\dot{\delta }f\right]}^{T}$$

(27) $$Z_{\mathrm{coh}}={\left[I_{E}\;Q_{E}\;I_{P}\;Q_{P}\;I_{L}\;Q_{L}\right]}^{T}$$

The state transition matrix of coherent pre-filter is written as follows:

(28) $$A_{\mathrm{coh}}=\left[\begin{array}{ccccc} 0 & 0 & 0 & 0 & 0\\ 0 & 1 & 0 & \beta \cdot T_{\mathrm{coh}} & 0\\ 0 & 0 & 1 & T_{\mathrm{coh}} & \frac{T_{\mathrm{coh}}{}^{2}}{2}\\ 0 & 0 & 0 & 1 & T_{\mathrm{coh}}\\ 0 & 0 & 0 & 0 & 1 \end{array}\right]$$

The process noise vector of coherent pre-filter is:

(29) $$w_{2}={\left[w_{A}\;w_{\mathrm{code}}\;w_{\mathrm{clock}}\;w_{\mathrm{drift}}\;w_{\mathrm{accel}}\right]}^{T}$$

where $w_{A}$ is the process noise for the signal amplitude.

The corresponding covariance matrix of the system model for coherent pre-filter is given by:

(30) $$Q_{\mathrm{coh}}=E[w_{2}w_{2}{}^{T}]$$

The measurement vector is a nonlinear function of state variables for coherent pre-filter. Thus, a Jacobian matrix is constructed to accomplish the correction in EKF. Detailed discussion of Jacobian matrix construction for coherent pre-filter is provided in a great deal of literature, and will not be shown here [13,17,19,20]. The observation noise variance of coherent pre-filter can be calculated as Equation (5), as long as the *C*/*N*_0_ estimation is available.

## 3. Enhanced Non-Coherent Pre-Filter Design

As discussed in Section 2, the non-coherent pre-filter takes the ATAN/ANAT2/NC-EMLE discriminator outputs as observations. However, there are three problems when we implement the non-coherent pre-filter. Firstly, as will be shown in the following parts, the Equations (18) and (19) are not accurate enough to calculate estimation noise variances of ATAN/ATAN2 discriminator in weak signal condition. Secondly, the estimation noise variance of NC-EMLE discriminator is difficult to express in analytical form, and thus is difficult to calculate. These two aspects hinder the correct setup of an observation noise variance matrix for a non-coherent pre-filter. Thirdly, the working range of a non-coherent pre-filter for carrier phase error estimation is constrained to (−0.25 cycle, 0.25 cycle) because of the limited effective working range of the ATAN discriminator. To address these three problems, this section will first analyze estimation noise variances of the ATAN/ANAT2/NC-EMLE discriminator through Monte Carlo simulation and then discuss the application of FDE structure in non-coherent pre-filter design.

As discussed in Section 2, the non-coherent pre-filter takes the ATAN/ANAT2/NC-EMLE discriminator outputs as observations. However, there are three problems when we implement the non-coherent pre-filter. Firstly, as will be shown in the following parts, the Equations (18) and (19) are not accurate enough to calculate estimation noise variances of ATAN/ATAN2 discriminator in weak signal condition. Secondly, the estimation noise variance of NC-EMLE discriminator is difficult to express in analytical form, and thus is difficult to calculate. These two aspects hinder the correct setup of an observation noise variance matrix for a non-coherent pre-filter. Thirdly, the working range of a non-coherent pre-filter for carrier phase error estimation is constrained to (−0.25 cycle, 0.25 cycle) because of the limited effective working range of the ATAN discriminator. To address these three problems, this section will first analyze estimation noise variances of the ATAN/ANAT2/NC-EMLE discriminator through Monte Carlo simulation and then discuss the application of FDE structure in non-coherent pre-filter design.

### 3.1. Observation Noise Characteristics Analysis of Non-Coherent Pre-Filter

Considering the complexity of the probability distribution of discriminator’s estimation noise, Monte Carlo simulation is introduced as the analysis tool. Roughly 40,000 groups of correlation values are simulated as Equation (4). Each group of correlation values contains six values, which are enough to execute the ATAN/ ATAN2/NC-EMLE discriminator algorithms. The real tracking error, *T*_coh_, *C*/*N*_0_ can be preset before the discriminator works. The values of estimation noise are calculated by subtracting the discriminator’s outputs from real tracking error. Thus, it is convenient for us to study the estimation noise characteristics of the ATAN/ATAN2/NC-EMLE discriminator in different *T*_coh_ and *C*/*N*_0_ without executing the intact tracking loop algorithms. Here we consider the discriminator and pre-filter as estimators, just a part of the tracking loops.

It also should be noted that the distribution of estimation noise of the ATAN/ATAN2/NC-EMLE discriminator changes with tracking error changing according to Equations (15)–(17). However, in this paper, we only analyze the condition where tracking error is zero, because this condition will simplify the analysis and will always be met when the tracking loop is in locking state.

**Figure 2:**
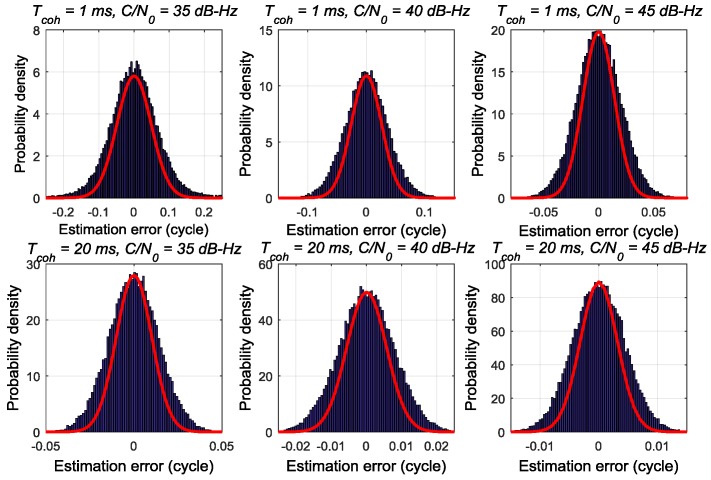
The estimation noise distribution of ATAN discriminator in different *T*_coh_ and *C*/*N*_0_.

**Figure 3:**
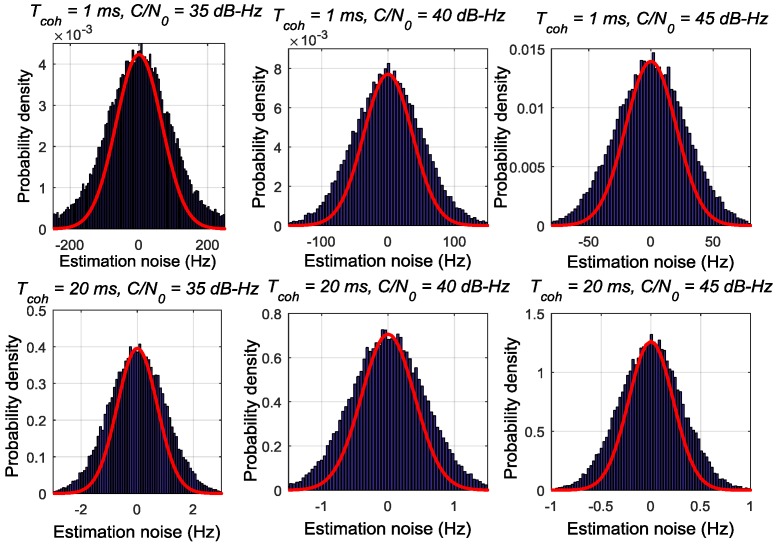
The estimation noise distribution of ATAN2 discriminator in different *T*_coh_ and *C*/*N*_0_.

**Figure 4:**
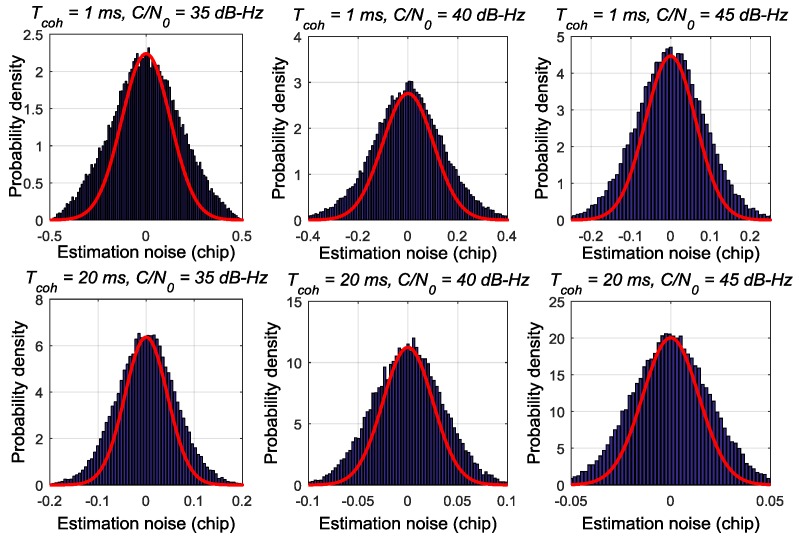
The estimation noise distribution of NC-EMLE discriminator in different *T*_coh_ and *C*/*N*_0_.

Figure 2, Figure 3 and Figure 4 respectively depict the estimation noise distribution of the ATAN/ATAN2/NC-EMLE discriminator in different *C*/*N*_0_ and *T*_coh_. The blue histograms in these figures indicate the statistical results of estimation noise. The statistical results have been divided by the number of simulated I/Q correlation groups so that units of y-axis can be transformed to probability density. The red lines in these figures represent the shape of Gaussian probability density distribution function whose mean and variance are same as those of corresponding statistical results. These figures imply that there is an obvious difference between the actual estimation noise distribution of the ATAN/ATAN2/NC-EMLE discriminator and a Gaussian distribution. That is, the estimation noise of the ATAN/ATAN2/NC-EMLE discriminator does not satisfy Gaussian distribution, which is consistent with the conclusion that can be given by Equations (15)–(17).

**Figure 5:**
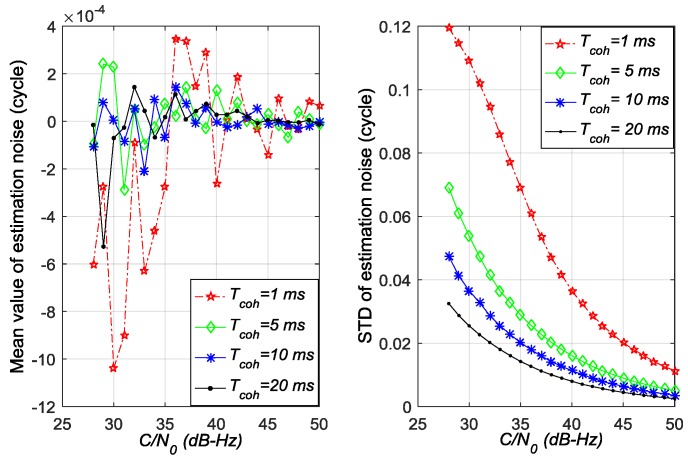
Mean values and standard deviations of estimation noise for ATAN discriminator in different *C*/*N*_0_ and *T*_coh_.

**Figure 6:**
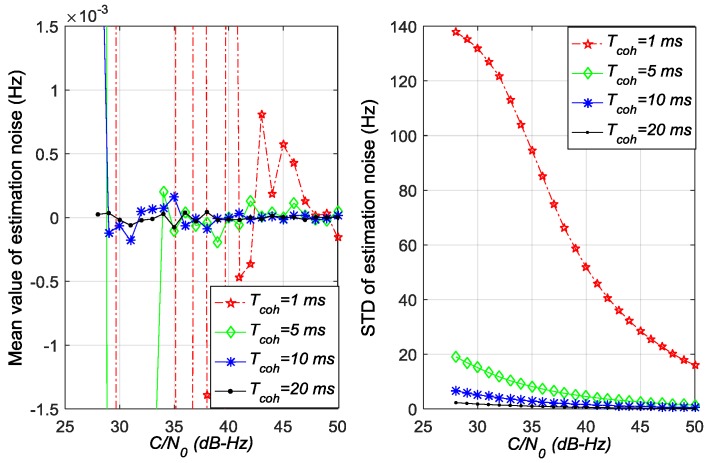
Mean values and standard deviations of estimation noise for ATAN2 discriminator in different *C*/*N*_0_ and *T*_coh_.

**Figure 7:**
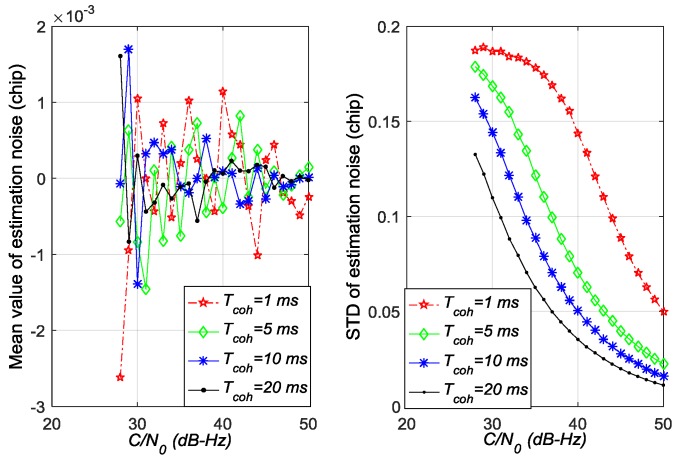
Mean values and standard deviations of estimation noise for NC-EMLE discriminator in different *C*/*N*_0_ and *T*_coh_ (d = 1 chip).

Figure 5, Figure 6 and Figure 7 show the mean values and standard deviation (STD) values of estimation noise for the ATAN/ATAN2/NC-EMLE discriminator in different *C*/*N*_0_ and *T*_coh_. On the one hand, the mean values of estimation noise, given by statistical analysis, are very close to zero in all conditions. On the other hand, these figures suggest that the variances of estimation noise of ATAN/ATAN2/NC-EMLE discriminator decrease with the *C*/*N*_0_, or *T*_coh_, or both increasing. Above all, these results are obtained by statistics of roughly 40,000 outputs of ATAN/ATAN2/NC-EMLE discriminator so that they are more close to the actual statistical characteristics of estimation noise.

**Figure 8:**
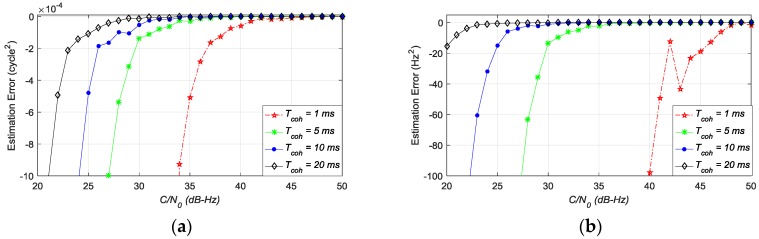
(**a**) The error of estimation noise variance of Equation (18) for ATAN discriminator; (**b**) The error of estimation noise variance of Equation (19) for ATAN2 discriminator.

Figure 8a,b shows the difference between the estimation noise variance obtained by Monte-Carlo simulation and estimation noise variance calculated by Equations (18) and (19). This figure indicates that these two equations can only provide relatively accurate estimation noise variances for the ATAN/ATAN2 discriminator when *C*/*N*_0_ is high or *T*_coh_ is long. However, these conditions cannot be satisfied at all times. Thus, Equations (18) and (19) are not accurate enough to calculate estimation noise variances of ATAN and ATAN2 discriminators. It also should be reemphasized that the estimation noise variance of the NC-EMLE discriminator is difficult to express as an analytical form.

**Figure 9:**
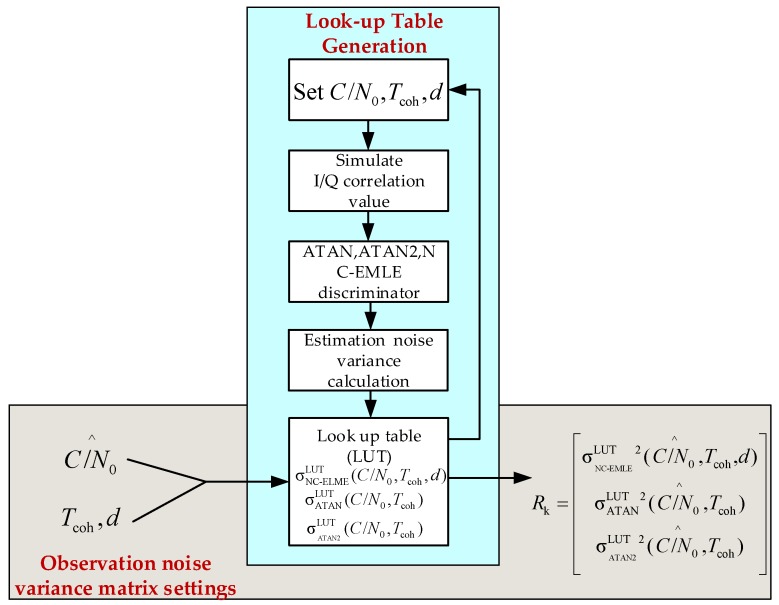
The generation and usage of look-up table.

Since the non-coherent takes the ATAN/ATAN2/NC-EMLE discriminator outputs as observations, the estimation noise variances of these discriminators are the elements of the observation noise variance matrix of the non-coherent pre-filter. However, these parameters cannot be calculated accurately according to the discussion above. In order to set the observation noise variance matrix correctly, it is a more realistic and reasonable to create a look-up table (LUT) that stores the relation among the *C*/*N*_0_, d, *T*_coh_, and estimation noise variances. Figure 9 shows the generation and usage of LUT, where $\hat{C/N_{0}}$ indicates the estimated *C*/*N*_0_, $R_{k}$ is the observation noise variance matrix. The LUT functions for the ATAN/ATAN2/NC-EMLE discriminator are expressed as $\sigma_{\mathrm{ATAN}}^{\mathrm{LUT}}(\cdot ,\cdot ,\cdot )$, $\sigma_{\mathrm{ATAN}2}^{\mathrm{LUT}}(\cdot ,\cdot ,\cdot )$, and $\sigma_{\mathrm{NC}-\mathrm{EMLE}}^{\mathrm{LUT}}(\cdot ,\cdot ,\cdot )$ respectively. As discussed in Section 3.1, the LUTs are obtained by Monte Carlo simulation. The *C*/*N*_0_, *T*_coh_, and d are first set to simulate I/Q correlation groups according to Equation (4). Then, we execute the ATAN/ATAN2/NC-EMLE discriminator algorithms according to Equations (12)–(14) and calculate the estimation noise variances for ATAN/ATAN2/NC-EMLE discriminator through the statistical analysis. After that, estimation noise variances are stored in the LUTs. The process discussed above is repeated until all the possible combinations of *C*/*N*_0_, *T*_coh_ and d are used. After these LUTs are constructed, the estimation noise variances of the ATAN/ATAN2/NC-EMLE discriminator can be found in these LUTs to set the observation noise variance matrix as long as the *C*/*N*_0_ is estimated correctly. The estimation of *C*/*N*_0_ is shown in Appendix C.

### 3.2. Implementing FDE Structure in the Non-Coherent Prefilter Design

Carrier phase tracking loop is the most vulnerable tracking loop in GNSS receivers. The vulnerability of carrier phase tracking partly comes from the limited working region of the ATAN discriminator. The effective working range of the ATAN discriminator is (−0.25 cycle, 0.25 cycle). If the input carrier phase error is beyond the effective working range, the ATAN discriminator would output the wrong estimation of carrier phase error. If this wrong estimation is entering the loop filter, the carrier NCO feedback commands would be adjusted toward the wrong direction and the PLL may lose lock for carrier phase.

As the non-coherent pre-filter takes ATAN discriminator outputs as observations, the limited work range of the ATAN discriminator also affects the non-coherent pre-filter. For recognizing and eliminating the wrong ATAN discriminator outputs, the FDE structure is introduced in non-coherent pre-filter.

**Figure 10:**
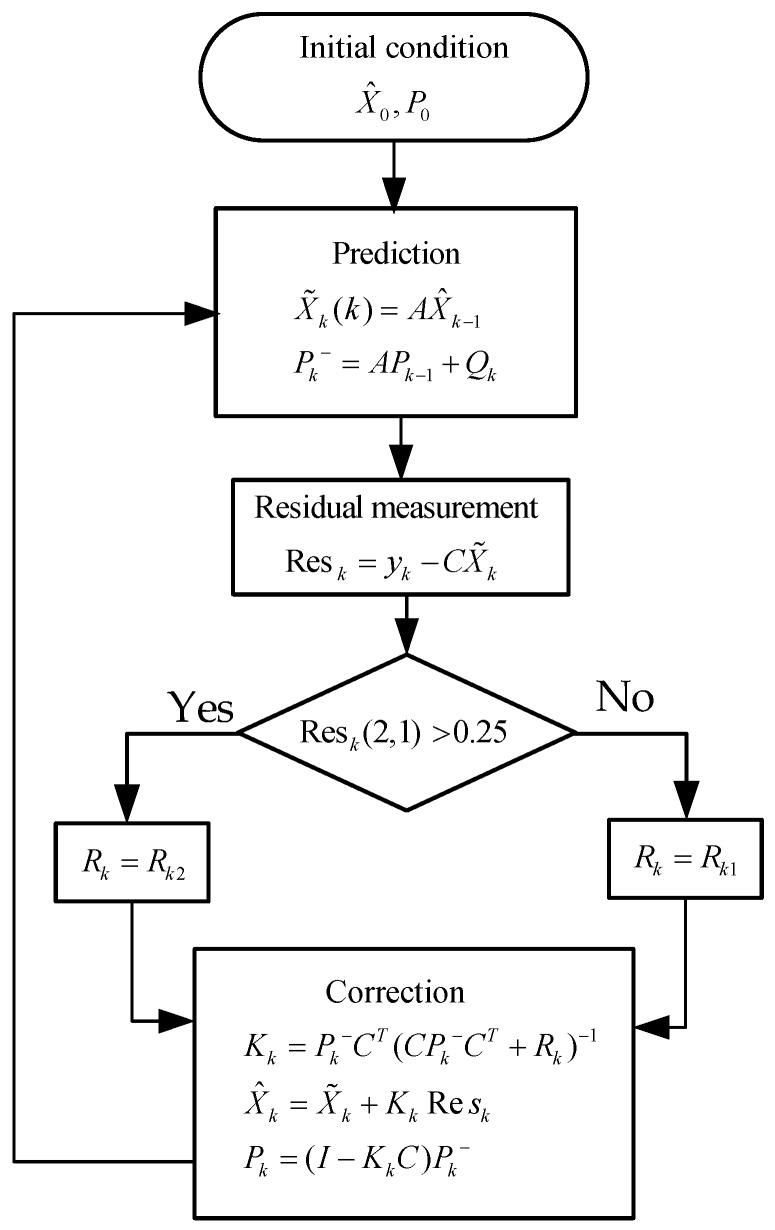
The fault detection and exclusion (FDE) structure for non-coherent pre-filter.

Figure 10 shows the operation flow chart of a non-coherent pre-filter with FDE structure. The symbols in Figure 10 are explained as follows: k represents the time stamps of Kalman filter, $\hat{X}$ is posteriori estimation for $X_{\mathrm{nonCoh}}$, $\tilde{X}$ is priori estimation for $X_{\mathrm{nonCoh}}$, $P_{k}{}^{-}$ is the covariance matrix of estimation error of $\tilde{X}$, $P_{k}$ is the covariance matrix of estimation error of $\hat{X}$, $R_{k}$ is the observation noise variance matrix, Res indicates the residuals of observations, Res(2,1) represents the 2nd row, 1st column element of vector Res and denotes the observation residual corresponding to the carrier phase error, K is the Kalman gain, I is unit matrix, A is the state transition matrix which is equal to $A_{\mathrm{nonCoh}}$, and C is the observation matrix which is equal to $C_{\mathrm{nonCoh}}$.

The FDE structure is aiming at reducing the weight of incorrect carrier phase error observations from ATAN discriminator outputs. This weight reduction is accomplished by adjusting the observation noise covariance matrix. If the input phase error is within the working range of the ATAN discriminator, Res(2,1)is very small. Under such a condition, the observation noise variance matrix is chosen as:

(31) $$R_{k1}=\left[\begin{array}{c} \sigma_{\mathrm{NC}-\mathrm{EMLE}}^{\mathrm{LUT}}{}^{2}(C/N_{0},T_{\mathrm{coh}},d)\\ \sigma_{\mathrm{ATAN}}^{\mathrm{LUT}}{}^{2}(C/N_{0},T_{\mathrm{coh}})\\ \sigma_{\mathrm{ATAN}2}^{\mathrm{LUT}}{}^{2}(C/N_{0},T_{\mathrm{coh}}) \end{array}\right]$$

If the input phase error is beyond the effective working range of the ATAN discriminator, the discriminator output would jump from −0.25 to 0.25, or from 0.25 to −0.25. Res(2,1) would also jump. This jump is easy to detect. In this paper, the threshold for detecting the jump is set to 0.25. After the jump of Res(2,1) is detected, the observation noise variance matrix is chosen as:

(32) $$R_{k2}=\left[\begin{array}{c} \sigma_{\mathrm{NC}-\mathrm{EMLE}}^{\mathrm{LUT}}{}^{2}(C/N_{0},T_{\mathrm{coh}},d)\\ 10000\\ \sigma_{\mathrm{ATAN}2}^{\mathrm{LUT}}{}^{2}(C/N_{0},T_{\mathrm{coh}}) \end{array}\right]$$

After the incorrect ATAN discriminator outputs are detected, their weight is decreased in the process of Kalman filtering. The carrier phase error estimation of the non-coherent pre-filter is mainly depending on the system model and ATAN2 discriminator output in this case. It also should be noted here that the non-coherent pre-filter cannot keep the correct estimation of carrier phase error with constant use of $R_{k2}$. The estimation error would accumulate with time because of observation noise of the ATAN2 discriminator and the error of system model.

## 4. Performance Evaluation

The performance evaluation presented here was carried out exploiting the simulated correlation value shown in Equation (4) and executing the discriminator/pre-filter algorithms discussed in Section 2 and Section 3. The tracking error dynamic and signal strength were varied in the carefully designed test scenes (i.e., test Scenes A, B, C, D) in order to evaluate the algorithms comprehensively. The algorithms evaluated in this paper include ATAN/ATAN2/NC-EMLE discriminators, the coherent pre-filter, and the enhanced non-coherent pre-filter. For the sake of brevity, the coherent pre-filter will be abbreviated to “Coh”, and the enhanced non-coherent pre-filter abbreviated to “Non-coh”. The metric used for evaluation is the root mean square (RMS) value of estimation error.

The common non-coherent pre-filter without FDE is not chosen as a method to be evaluated. There are two reasons why we did not do this. Firstly, in previous work, the observation noise variance matrix of the non-coherent pre-filter is determined by filter tuning [13,16]. Secondly, the advantage of the enhanced non-coherent pre-filter over ordinary non-coherent pre-filter has been analyzed in Section 3.2. We would also discuss this advantage in the following experiments.

### 4.1. Carrier Phase Error/Code Phase Error Step Scene

In test scene A, the *C*/*N*_0_ of the GNSS signal was set to 45 dB-Hz. There was a step input for carrier phase error at 1500 milliseconds (ms), and for code phase error at 2000 ms. *T*_coh_ was set to 1 ms, d set to 1 chip. This test was used to evaluate the performance of the ATAN/ATAN2/NC-EMLE discriminator and pre-filter in normal condition.

**Figure 11:**
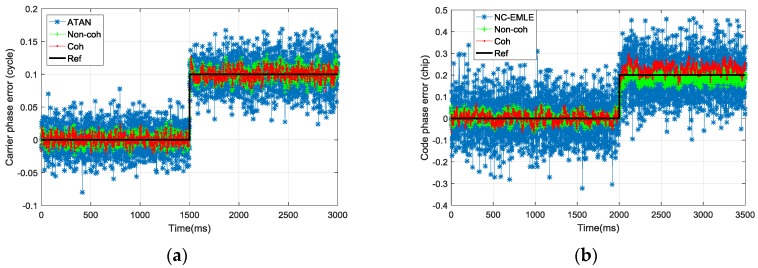
(**a**) The carrier phase error estimation in test scene A; (**b**) The code phase error estimation in test scene A.

Figure 11 shows the tracking error estimation results in test scene A. The black line represents the real tracking error when we simulated I/Q correlation groups. As expected, the coherent/non-coherent pre-filter were able to estimate the carrier phase error and code phase error accurately. The convergence time of pre-filter was less than 200 ms when step-input occurred. The estimations of pre-filter were less noisy than that of the ATAN/ATAN2/NC-EMLE discriminator. The estimation accuracy of the coherent pre-filter for tracking error was slightly higher than that of the non-coherent pre-filter, as shown in Table 2.

### 4.2. Constant Carrier Frequency Error Scene

In test scene B, the carrier frequency error was set to 10 Hz, which indicates the carrier phase error would accumulate linearly with time. *T*_coh_ was set to 1 ms, while the *C*/*N*_0_ was set to 45 dB-Hz, d was set to 1 chip. This test was aimed at showing the power of FDE in the non-coherent pre-filter in the scene where the carrier phase error is beyond the effective working range of the ATAN discriminator.

**Figure 12:**
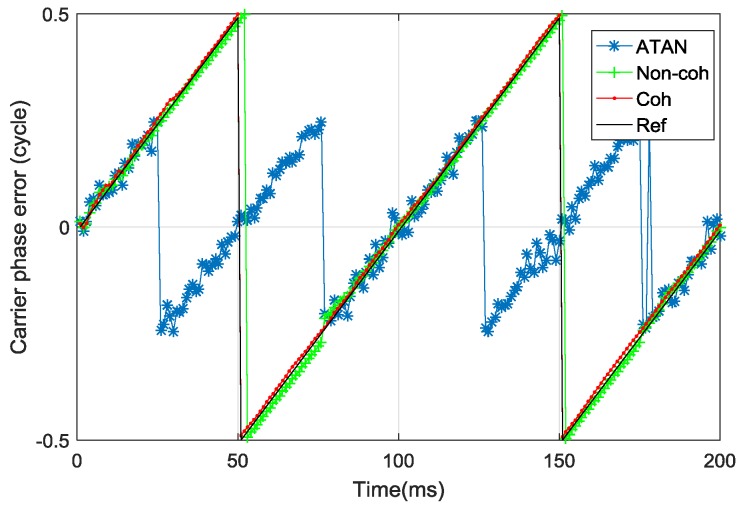
Carrier phase error estimation in test scene B.

Figure 12 shows the carrier phase error estimation in test scene B. The black line represents the dynamic change of real carrier phase error. It can be seen that the carrier phase error estimated by the ATAN discriminator would be totally wrong when the real carrier phase error is outside the range of (−0.25 cycle, 0.25 cycle). The ATAN discriminator outputs are the observations of non-coherent pre-filter, which indicates that the effective working range of the non-coherent pre-filter without FDE for carrier phase error estimation is also (−0.25 cycle, 0.25 cycle). If these false observations are not eliminated or their weight is not reduced, the non-coherent pre-filter’s estimation error will increase. Through the FDE structure, the work range of the non-coherent pre-filter for carrier phase error estimation is extended from (−0.25 cycle, 0.25 cycle) to (−0.5 cycle, 0.5 cycle). The enhanced non-coherent pre-filter has comparable performance as coherent pre-filter in test scene B, as shown in Table 3.

### 4.3. Varying Carrier Frequency Error Scene

In test scene C, the carrier frequency error was varying as Equation (33), where K=20 Hz; α=−1; ω=−2π rad/s. *T*_coh_ was set to 1 ms while *C*/*N*_0_ was set to 45 dB-Hz, and d was set to 1 chip. This configuration is closer to what really happened in the signal tracking loop when the GNSS receiver was faced with high dynamic motion. The convergence with oscillation of tracking error often occurs in a highly dynamic scene. This test is designed to evaluate the estimation accuracies of discriminator and pre-filter algorithms in a highly dynamic scenario.

(33) $$\delta f(t)=K\cdot e^{\alpha t}\cdot \sin(\omega t)$$

**Figure 13:**
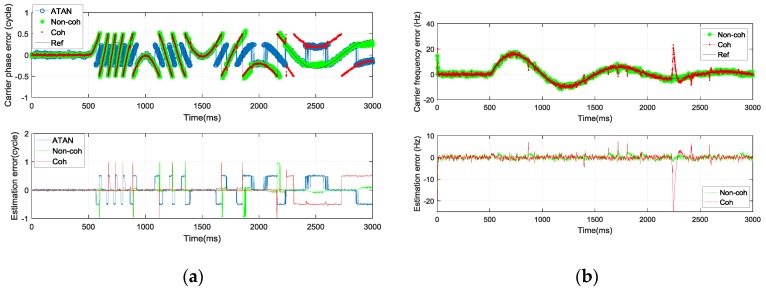
(**a**) The carrier phase error estimation in test scene C; (**b**) The carrier frequency error estimation in test scene C.

Figure 13 shows the carrier phase/frequency tracking error estimation. Similarly, there are still estimation outliers for the ATAN discriminator when the real carrier phase error exceeds the ATAN discriminator’s effective working region. There are also some estimation outliers for the pre-filter. After 2250 ms, the coherent pre-filter cannot estimate the carrier phase error correctly. The estimation outliers of the coherent pre-filter appear because the navigation bits cannot be estimated correctly according to the sign of *I*_E_ in test scene C. Thus, the state variables of the coherent pre-filter converge to the wrong value. Even though are there estimation outliers in the non-coherent pre-filter for carrier phase tracking error estimation, it can estimate the tracking error correctly and accurately most of the time. The outliers in the non-coherent pre-filter appear because of the constant use of $R_{k2}$, as discussed in Section 3.2. By the time real carrier phase error approaches −0.5 cycle or 0.5 cycle, the non-coherent pre-filter has worked with $R_{k2}$ for a very long time. Thus, the estimation error is relatively large. The FDE structure cannot switch the $R_{k}$ from $R_{k2}$ to $R_{k1}$ quickly in this condition.

The number of estimation outliers of the non-coherent pre-filter is obviously less than that of the coherent pre-filter. Thus, the robustness of the non-coherent pre-filter is stronger than that of the coherent pre-filter in a high tracking error dynamic scene. The RMS values of the estimation error of the three algorithms in scene C are shown in Table 4. It can be concluded that the estimation accuracy of the enhanced non-coherent pre-filter outperforms the ATAN/ATAN2/NC-EMLE discriminator and coherent pre-filter in high tracking error dynamic change condition, which often occurs when the tracking loop suffers from high dynamic movement of GNSS receivers. Compared with the coherent pre-filter, the enhanced non-coherent pre-filter provides accuracy improvements of 41.6%, 46.4%, and 50.36% for carrier phase error, carrier frequency error, and code phase error estimation, respectively, in test scene C.

### 4.4. Estimation Accuracy Evaluation Under Various C/N_0_

In test scene D, the estimation accuracies of three algorithms were evaluated in different *C*/*N*_0_. *T*_coh_ was set to 20 ms in order to test the algorithms in weak signal condition. d was set to 1 chip. The tracking error was set to zero, assuming that the tracking loop is in locking state.

**Figure 14:**
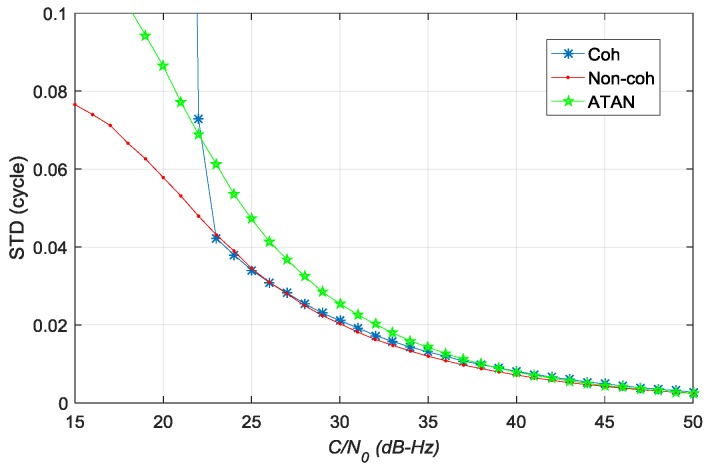
The standard deviation (STD) values of estimation error of carrier phase error in different *C*/*N*_0_.

Figure 14 shows the STD values of estimation error for carrier phase error. The accuracy comparisons are shown in Table 5. The estimation accuracy of pre-filter for carrier phase error is better than that of the traditional ATAN discriminator. The non-coherent pre-filter outperforms the other two algorithms when $C/N_{0}\in$ [26,50]∪[15,23]. The coherent pre-filter outperforms the others when $C/N_{0}\in [23,28]$.

**Figure 15:**
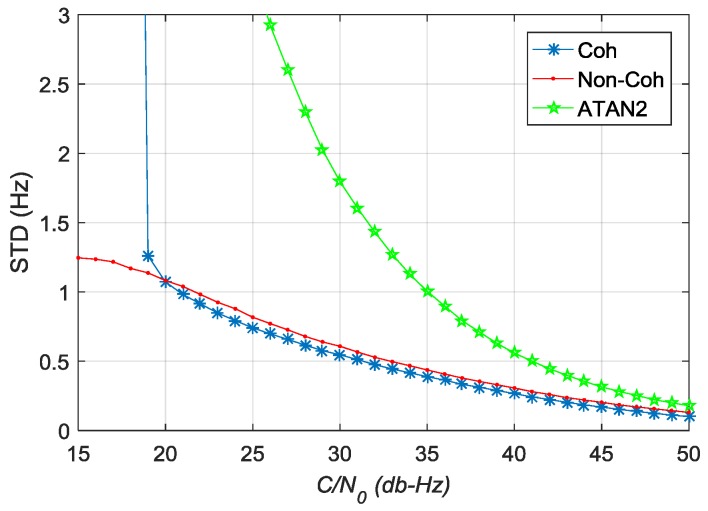
The STD values of estimation error of carrier frequency error in different *C*/*N*_0_.

Figure 15 shows the STD values of estimation error for carrier frequency error. The pre-filter outperforms the ATAN2 discriminator in different values of *C*/*N*_0_. When *C*/*N*_0_ is greater than 20 dB-Hz, the estimation accuracy of coherent pre-filter for carrier frequency error is higher than that of non-coherent pre-filter. When *C*/*N*_0_ is less than 20 dB-Hz, the estimation accuracy of coherent pre-filter reduces seriously and the performance of non-coherent pre-filter is better than that of the coherent pre-filter.

**Figure 16:**
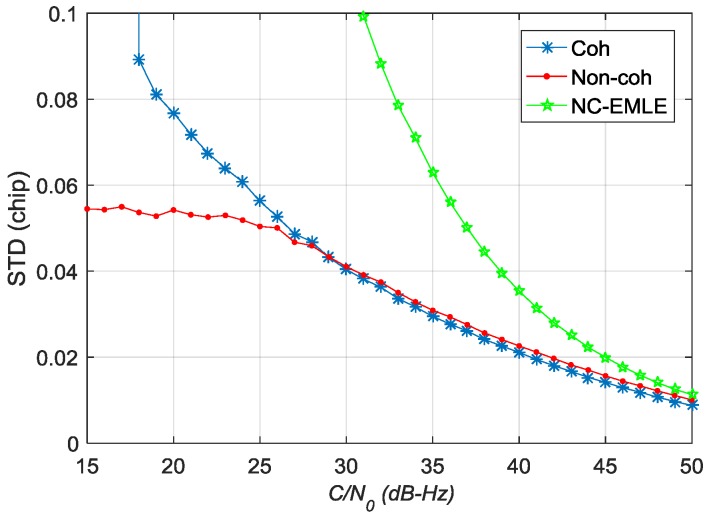
The STD values of estimation error of code phase error in different *C*/*N*_0_.

Figure 16 shows the STD values of estimation error for code phase error. Similarly, the pre-filter outperforms NC-EMLE discriminator in different *C*/*N*_0_. When *C*/*N*_0_ is greater than 28.8 dB-Hz, the estimation accuracy of the coherent pre-filter for code phase error is higher than that of the non-coherent pre-filter. When *C*/*N*_0_ is less than 28.8 dB-Hz, the estimation accuracy of the coherent pre-filter for code phase error reduces seriously and the non-coherent pre-filter outperforms the coherent pre-filter.

Overall, the tracking error estimated by coherent/non-coherent pre-filter is less noisy than that estimated by the ATAN/ATAN2/NC-EMLE discriminator. The non-coherent pre-filter outperforms the coherent pre-filter for tracking error estimation in weak signal or highly dynamic scene. The non-coherent pre-filter also outperforms the coherent pre-filter for carrier phase error estimation in strong signal condition.

## 5. Conclusions and Future Work

This paper presents an enhanced non-coherent pre-filter design. Firstly, the observation noise variance of non-coherent pre-filter is analyzed through Monte Carlo Simulation. The LUTs are subsequently created to store the relationship among *T*_coh_, *C*/*N*_0_, and the estimation noise variances of the ATAN/ATAN2/NC-EMLE discriminator. The LUTs provide a method to set the observation noise variance matrix of non-coherent pre-filter correctly according to estimated *C*/*N*_0_. Secondly, a simple FDE structure is introduced to overcome the inherent deficiency of the ATAN discriminator, whose outputs are observations of the non-coherent pre-filter. Through the FDE structure, the effective estimation range of the non-coherent pre-filter for carrier phase error extends from (−0.25 cycle, 0.25 cycle) to (−0.5 cycle, 0.5 cycle). This is a significant improvement that makes the non-coherent pre-filter outperform the coherent pre-filter in highly dynamic scenes.

The performance of the proposed enhanced non-coherent pre-filter is compared with traditional ATAN/ATAN2/NC-EMLE discriminators and the coherent pre-filter in carefully designed scenarios. The estimation accuracy and robustness of the enhanced non-coherent pre-filter outperform the traditional discriminator and coherent pre-filter in weak signal condition and high tracking error dynamic scene. Additionally, the estimation accuracy of the enhanced non-coherent pre-filter for carrier phase error outperforms the coherent pre-filter in strong signal scenes. The best operating conditions of the coherent/non-coherent pre-filter for tracking error estimation are discussed in detail in the performance evaluation section. This can be the basis of hybrid pre-filter design, where tracking error would be estimated by coherent and non-coherent pre-filter simultaneously.

The accuracy evaluation of discriminator and pre-filter algorithms is based on the simulated I/Q correlation signal in this paper. It is also necessary to evaluate the accuracy improvements which can be obtained by using the enhanced non-coherent pre-filter in the aspect of position fixing and velocity determination. Besides, the tracking error estimated by pre-filter is less noisy than that of the ATAN/ATAN2/NC-EMLE discriminator. If the state variables of the pre-filter, instead of discriminator outputs, are used to generate carrier/code NCO feedback command, the tracking loop is able to track weaker signal or higher signal dynamic, or both. However, it is still a question how to generate NCO command according to state variables of pre-filter. The performance of the pre-filter can be further improved by adjusting the system noise co-variance matrix, which is not discussed in this paper. These three aspects can be the future research orientations.

## Figures and Tables

**Table 1:** The mathematical model of coherent/non-coherent integration results.

Signal	Useful Signal	Noise	Noise Distribution
$I_{E}$	$A_{i}\cdot D_{i}\cdot R(\delta \tau_{i}-\frac{d}{2})\sin c(\delta f_{i}T_{\mathrm{coh}})\cos(\bar{\delta \varphi_{i}})$	$n_{\mathrm{IE}}$	Gaussian white noise $N(0,\sigma_{\mathrm{noise}}{}^{2})$
$Q_{E}$	$A_{i}\cdot D_{i}\cdot R(\delta \tau_{i}-\frac{d}{2})\sin c(\delta f_{i}T_{\mathrm{coh}})\sin(\bar{\delta \varphi_{i}})$	$n_{\mathrm{QE}}$	
$I_{P}$	$A_{i}\cdot D_{i}\cdot R(\delta \tau_{i})\sin c(\delta f_{i}T_{\mathrm{coh}})\cos(\bar{\delta \varphi_{i}})$	$n_{\mathrm{IP}}$	
$Q_{P}$	$A_{i}\cdot D_{i}\cdot R(\delta \tau_{i})\sin c(\delta f_{i}T_{\mathrm{coh}})\sin(\bar{\delta \varphi_{i}})$	$n_{\mathrm{QP}}$	
$I_{L}$	$A_{i}\cdot D_{i}\cdot R(\delta \tau_{i}+\frac{d}{2})\sin c(\delta f_{i}T_{\mathrm{coh}})\cos(\bar{\delta \varphi_{i}})$	$n_{\mathrm{IL}}$	
$Q_{L}$	$A_{i}\cdot D_{i}\cdot R(\delta \tau_{i}+\frac{d}{2})\sin c(\delta f_{i}T_{\mathrm{coh}})\sin({\bar{\delta \varphi }}_{i})$	$n_{\mathrm{QL}}$	
$E^{2}$	$A^{2}{\left|\sin c(\delta fT_{\mathrm{coh}})\right|}^{2}\;\sum_{j=1}^{N_{\mathrm{nc}}}R^{2}(\delta \tau_{j}-\frac{d}{2})$	$N_{\mathrm{nc}}n_{E^{2}}$	White noise with unknown distribution
$P^{2}$	$A^{2}{\left|\sin c(\delta fT_{\mathrm{coh}})\right|}^{2}\;\sum_{j=1}^{N_{\mathrm{nc}}}R^{2}(\delta \tau_{j})$	$N_{\mathrm{nc}}n_{P^{2}}$	
$L^{2}$	$A^{2}{\left|\sin c(\delta fT_{\mathrm{coh}})\right|}^{2}\;\sum_{j=1}^{N_{\mathrm{nc}}}R^{2}(\delta \tau_{j}+\frac{d}{2})$	$N_{\mathrm{nc}}n_{L^{2}}$	
E	$A\left|\sin c(\delta fT_{\mathrm{coh}})\right|\;\sum_{j=1}^{N_{\mathrm{nc}}}R(\delta \tau_{j}-\frac{d}{2})$	$N_{\mathrm{nc}}n_{E}$	White noise with unknown distribution
P	$A\left|\sin c(\delta fT_{\mathrm{coh}})\right|\;\sum_{j=1}^{N_{\mathrm{nc}}}R(\delta \tau_{j})$	$N_{\mathrm{nc}}n_{P}$	
L	$A\left|\sin c(\delta fT_{\mathrm{coh}})\right|\;\sum_{j=1}^{N_{\mathrm{nc}}}R(\delta \tau_{j}+\frac{d}{2})$	$N_{\mathrm{nc}}n_{L}$	

**Table 2:** Performance comparison of tracking error estimation methods in test scene A.

Tracking Error Estimation Method	Root Mean Square of Estimation Error for Tracking Error		
	Carrier Phase Error (cycle)	Carrier Frequency Error (Hz)	Code Phase Error (chip)
Discriminator	0.023002	32.4045	0.1715
Coherent pre-filter	0.007829	0.7464	0.0237
Non-coherent pre-filter	0.009757	0.8930	0.0322

**Table 3:** Performance comparison of tracking error estimation methods in test scene B.

Tracking Error Estimation Method	Root Mean Square of Estimation Error for Tracking Error		
	Carrier Phase Error (cycle)	Carrier Frequency Error (Hz)	Code Phase Error (chip)
Discriminator	0.35206	28.2365	0.08783
Coherent pre-filter	0.05035	0.40724	0.00723
Non-coherent pre-filter	0.05475	0.43523	0.00813

**Table 4:** Performance comparison of tracking error estimation methods in test scene C.

Tracking Error Estimation Method	Root Mean Square of Estimation Error for Tracking Error		
	Carrier phase Error (cycle)	Carrier Frequency Error (Hz)	Code Phase Error (chip)
Discriminator	0.30656	27.81829	0.08758
Coherent pre-filter	0.26179	2.00818	0.07386
Non-coherent pre-filter	0.15270	1.04078	0.03666

**Table 5:** Accuracy comparison of carrier phase error estimation.

*C*/*N*_0_ (dB-Hz)	Accuracy Comparison
[38.7, 50]	Non-coh > ATAN > Coh
[26, 38.7]	Non-coh > Coh > ATAN
[23, 26]	Coh > Non-coh > ATAN
[15, 23]	Non-coh > ATAN > Coh

## Appendix A. The Derivation and Definition of *P*_cross_, *P*_dot_, *B*(*n*), *C*_1_(*n*), *C*_2_(*n*)

The coherent integration in contiguous epoch, *n* and *n* − 1, is redefined as:

(A1) $$I_{P}(n)=A\cdot R(\delta \tau_{n})\sin c(\delta f_{n}T_{\mathrm{coh}})\cos({\bar{\delta \varphi }}_{n})+n_{\mathrm{IP}}$$

(A2) $$Q_{P}(n)=A\cdot R(\delta \tau_{n})\sin c(\delta f_{n}T_{\mathrm{coh}})\sin({\bar{\delta \varphi }}_{n})+n_{\mathrm{QP}}$$

(A3) $$I_{P}(n-1)=A\cdot R(\delta \tau_{n-1})\sin c(\delta f_{n-1}T_{\mathrm{coh}})\cos({\bar{\delta \varphi }}_{n-1})+n_{\mathrm{IP}}$$

(A4) $$Q_{P}(n-1)=A\cdot R(\delta \tau_{n-1})\sin c(\delta f_{n-1}T_{\mathrm{coh}})\sin({\bar{\delta \varphi }}_{n-1})+n_{\mathrm{QP}}$$

After that, the *P*_cross_ and *P*_dot_ contained in ATAN2 discriminator algorithm are defined as Equations (A5) and (A6).

(A5) $$P_{\mathrm{cross}}(n)=I_{P}(n-1)Q_{P}(n)+I_{P}(n)Q_{P}(n-1)$$

(A6) $$P_{\mathrm{dot}}(n)=I_{P}(n-1)I_{P}(n)+Q_{P}(n-1)Q_{P}(n)$$

Inserting Equations (A1)–(A4) into Equations (A5) and (A6), *P*_cross_ and *P*_dot_ can be expressed as:

(A7) $$P_{\mathrm{cross}}(n)=B(n)B(n-1)\sin(\delta \varphi_{n}-\delta \varphi_{n-1})+C_{1}(n)$$

(A8) $$P_{\mathrm{dot}}(n)=B(n)B(n-1)\cos(\delta \varphi_{n}-\delta \varphi_{n-1})+C_{2}(n)$$

where

(A9) $$B(n)=A\cdot R(\delta \tau_{n})\sin c(\delta f_{n}T_{\mathrm{coh}})$$

(A10) $$\begin{array}{ll} C_{1}(n) & =n_{\mathrm{IP}}B(n)\sin{\bar{\delta \varphi }}_{n}+n_{\mathrm{QP}}B(n-1)\cos{\bar{\delta \varphi }}_{n-1}+n_{\mathrm{QP}}B(n)\cos{\bar{\delta \varphi }}_{n}+n_{\mathrm{IP}}B(n-1)\sin{\bar{\delta \varphi }}_{n-1}+2n_{\mathrm{IP}}n_{\mathrm{QP}}\\ & =n_{\mathrm{IP}}[B(n)\sin{\bar{\delta \varphi }}_{n}+B(n-1)\cos{\bar{\delta \varphi }}_{n-1}+B(n)\cos{\bar{\delta \varphi }}_{n}+B(n-1)\sin{\bar{\delta \varphi }}_{n-1}]+2n_{\mathrm{IP}}{}^{2} \end{array}$$

(A11) $$\begin{array}{ll} C_{2}(n) & =n_{\mathrm{IP}}B(n)\cos{\bar{\delta \varphi }}_{n}+n_{\mathrm{IP}}B(n-1)\cos{\bar{\delta \varphi }}_{n-1}+n_{\mathrm{QP}}B(n)\sin{\bar{\delta \varphi }}_{n}+n_{\mathrm{QP}}B(n-1)\sin{\bar{\delta \varphi }}_{n-1}+n_{\mathrm{IP}}{}^{2}+n_{\mathrm{QP}}{}^{2}\\ & =n_{\mathrm{IP}}[B(n)\sin{\bar{\delta \varphi }}_{n}+B(n-1)\cos{\bar{\delta \varphi }}_{n-1}+B(n)\cos{\bar{\delta \varphi }}_{n}+B(n-1)\sin{\bar{\delta \varphi }}_{n-1}]+2n_{\mathrm{IP}}{}^{2} \end{array}$$

It should be noted here that the noise terms, $n_{\mathrm{IP}}$ and $n_{\mathrm{QP}}$, are dependent and of the same distribution when we derive Equations (A10) and (A11).

## Appendix B. Derivation of Estimation Noise Variance of ATAN2 FLL Discriminator

In order to derive the estimation noise variance of the ATAN2 discriminator, we assume this estimation noise is white noise. Hence, Equation (A12) is established as follows:

(A12) $$\begin{array}{l} E(n_{\mathrm{ATAN}})=0\\ D(n_{\mathrm{ATAN}})=E({\left[n_{\mathrm{ATAN}}-E(n_{\mathrm{ATAN}})\right]}^{2})=E(n_{\mathrm{ATAN}}{}^{2})=\sigma_{\mathrm{ATAN}}{}^{2}\\ E[n_{\mathrm{ATAN}}(n)n_{\mathrm{ATAN}}(n-1)]=0 \end{array}$$

where $E(n_{\mathrm{ATAN}})$ is the mean value of $n_{\mathrm{ATAN}}$. $D(n_{\mathrm{ATAN}})$ is the variance of $n_{\mathrm{ATAN}}$. According to Reference [9], the ATAN2 algorithm is equivalent to Equation (A13):

(A13) $$\delta f_{\mathrm{ANAN}2}(n)=\frac{\delta \varphi_{\mathrm{ANAN}}(n)-\delta \varphi_{\mathrm{ATAN}}(n-1)}{T_{\mathrm{coh}}}$$

Plug Equation (15) into Equation (A13), the $\delta f_{\mathrm{ANAN}2}(n)$ can be reformulated as follows:

(A14) $$\begin{array}{l} \delta f_{\mathrm{ANAN}2}(n)=\frac{\delta \varphi (n)-\delta \varphi (n-1)}{T_{\mathrm{coh}}}+\frac{n_{\mathrm{ATAN}}(n)-n_{\mathrm{ATAN}}(n-1)}{T_{\mathrm{coh}}}\\ =\delta f+\frac{n_{\mathrm{ATAN}}(n)-n_{\mathrm{ATAN}}(n-1)}{T_{\mathrm{coh}}}=\delta f+n_{\mathrm{ATAN}2} \end{array}$$

where $n_{\mathrm{ATAN}2}=\frac{n_{\mathrm{ATAN}}(n)-n_{\mathrm{ATAN}}(n-1)}{T_{\mathrm{coh}}}$.

This conclusion gives us an instruction that we can derive $D(n_{\mathrm{ATAN}2})$ according to the statistical property of $n_{\mathrm{ATAN}}$. $D(n_{\mathrm{ATAN}2})$ is calculated as follows:

(A15) $$D(n_{\mathrm{ATAN}2})=E({\left[n_{\mathrm{ATAN}2}-E(n_{\mathrm{ATAN}2})\right]}^{2})=E(n_{\mathrm{ANAT}2}{}^{2})-E^{2}(n_{\mathrm{ATAN}2})$$

The first term of $D(n_{\mathrm{ATAN}2})$ can be derived as:

(A16) $$E(n_{\mathrm{ANAT}2}{}^{2})=E[{\left(\frac{n_{\mathrm{ATAN}}(n)-n_{\mathrm{ATAN}}(n-1)}{T_{\mathrm{coh}}}\right)}^{2}]=\frac{2}{T_{\mathrm{coh}}{}^{2}}E(\varphi^{2}{}_{\mathrm{ATAN}})$$

The second term of $D(n_{\mathrm{ATAN}2})$ is

(A17) $$E^{2}(n_{\mathrm{ATAN}2})=E^{2}(\frac{n_{\mathrm{ATAN}}(n)-n_{\mathrm{ATAN}}(n-1)}{T_{\mathrm{coh}}})=0$$

Plug Equations (A16)–(A18) into Equation (A15), we can get the analytical solution of $D(n_{\mathrm{ATAN}2})$ as Equation (A18).

(A18) $$D(n_{\mathrm{ATAN}2})=\frac{2}{T_{\mathrm{coh}}{}^{2}}E(\varphi^{2}{}_{\mathrm{ATAN}})=\frac{2}{T_{\mathrm{coh}}{}^{2}}\sigma_{\mathrm{ATAN}}{}^{2}={\left(\frac{1}{2\pi }\right)}^{2}\frac{1}{c/n_{0}\cdot T_{\mathrm{coh}}{}^{3}}(1+\frac{1}{c/n_{0}\cdot T_{\mathrm{coh}}})$$

## Appendix C. *C*/*N*_0_ Estimation of GNSS Signals

The power ratio method is used to estimate *C*/*N*_0_ in this paper, which involves the comparison between wideband power *P*_w_ and narrowband power *P*_n_. The estimation of *C*/*N*_0_ is shown as follows:

(A19) $$P_{w}=\sum_{n=1}^{M}\left(I_{P}{\left(n\right)}^{2}+Q_{P}{\left(n\right)}^{2}\right)$$

(A20) $$P_{n}=(\sum_{n=1}^{M}I_{P}(n){)}^{2}+(\sum_{n=1}^{M}Q_{P}(n){)}^{2}$$

(A21) $$P_{n/w}=\frac{1}{N}\sum_{r=1}^{N}\frac{P_{N,r}}{P_{W,r}}$$

(A22) $$c/n_{0}=\frac{1}{T_{\mathrm{coh}}}(\frac{P_{n/w}-1}{M-P_{n/w}})$$

(A23) $$C/N_{0}=10\mathrm{lg}(c/n_{0})$$

where *M* and *N* are parameters of power ratio method, *c*/*n*_0_ is the carrier-to-noise density ratio in the unit of Hz, and *C*/*N*_0_ is the carrier-to-noise density ratio in the unit of dB-Hz.
